# Synergistic activity of pomegranate rind extract and Zn (II) against *Candida albicans* under planktonic and biofilm conditions, and a mechanistic insight based upon intracellular ROS induction

**DOI:** 10.1038/s41598-022-21995-7

**Published:** 2022-11-15

**Authors:** Vildan Celiksoy, Rachael L. Moses, Alastair J. Sloan, Ryan Moseley, Charles M. Heard

**Affiliations:** 1 School of Pharmacy and Pharmaceutical Sciences, Cardiff University, Cardiff, UK; 2 School of Dentistry, Cardiff University, Cardiff, UK; 3 Faculty of Medicine, Dentistry and Health Sciences, Melbourne Dental School, University of Melbourne, Parkville, VIC Australia

**Keywords:** Drug discovery, Microbiology, Plant sciences

## Abstract

*Candida albicans* (*C. albicans*) is an opportunistic pathogen, which causes superficial infection and can lead to mortal systemic infections, especially in immunocompromised patients. The incidence of *C. albicans* infections is increasing and there are a limited number of antifungal drugs used in treatment. Therefore, there is an urgent need for new and alternative antifungal drugs. Pomegranate rind extract (PRE) is known for its broad-spectrum antimicrobial activities, including against *C. albicans* and recently, PRE and Zn (II) have been shown to induce synergistic antimicrobial activity against various microbes. In this study, the inhibitory activities of PRE, Zn (II) and PRE in combination with Zn (II) were evaluated against *C. albicans.* Antifungal activities of PRE and Zn (II) were evaluated using conventional microdilution methods and the interaction between these compounds was assessed by in vitro checkerboard and time kill assays in planktonic cultures. The anti-biofilm activities of PRE, Zn (II) and PRE in combination with Zn (II) were assessed using confocal laser scanning microscopy, with quantitative analysis of biofilm biomass and mean thickness analysed using COMSTAT2 analysis. In addition, antimicrobial interactions between PRE and Zn (II) were assayed in terms reactive oxygen species (ROS) production by *C. albicans*. PRE and Zn (II) showed a potent antifungal activity against *C. albicans*, with MIC values of 4 mg/mL and 1.8 mg/mL, respectively. PRE and Zn (II) in combination exerted a synergistic antifungal effect, as confirmed by the checkerboard and time kill assays. PRE, Zn (II) and PRE and Zn (II) in combination gave rise to significant reductions in biofilm biomass, although only PRE caused a significant reduction in mean biofilm thickness. The PRE and Zn (II) in combination caused the highest levels of ROS production by *C. albicans,* in both planktonic and biofilm forms. The induction of excess ROS accumulation in *C. albicans* may help explain the synergistic activity of PRE and Zn (II) in combination against *C. albicans* in both planktonic and biofilm forms. Moreover, the data support the potential of the PRE and Zn (II) combination as a novel potential anti-*Candida* therapeutic system.

## Introduction

The prevalence of fungal infection has steadily increased as a result of the extensive use of hormones, immunosuppressants and broad-spectrum antibiotics^1^. *Candida* is a yeast-like fungus that normally lives on skin and inside the body such as in the mouth, throat, gut, and vagina, without causing any problems. However, uncontrolled proliferation leads to candidiasis, which is a broad term that refers to a group of infections affecting cutaneous, mucosal or deep-seated tissues^2,3^. Oral candidiasis can progress to severe stomatitis, which can lead to life-threatening bloodstream and tissue infections^4^. Intraocular *Candida* infection and subsequent ocular candidiasis can have devastating visual consequences^5^. *Candida* is also the causative agent of vulvovaginal candidiasis, which has the highest incidence of any single infectious diseases on the world^6^. Azole, polyenes, and echinocandins are currently the most often used medications to treat candidiasis^7^. However, the extensive use of these medications may exacerbate multidrug resistance and significantly diminish treatment efficacy^8,9^. In addition, side effects such as nephrotoxicity, hepatotoxicity, haemolytic anaemia, are limitations in the treatment of *Candida* infections^10,11^. As a result, novel antifungal drugs for the treatment of *Candida*-related infections are urgently needed.

Many natural compounds have applications as antifungal therapies, due to their potencies, abundant supplies and low toxicities^12^. The pomegranate, fruit of *Punica granatum* L., has a long folkloric history of treating infections, and antimicrobial activity has been reported in recent times against different microbes, including *C. albicans*^13–15^. It was shown that pomegranate peel/rind extract inhibit fungal growth by compromising the cell wall and the cytoplasmic membrane^16,17^. The antimicrobial activity of pomegranate extracts has been largely attributed to its polyphenolic content, in particular the hydrolysable ellagitannin, punicalagin, which is particularly abundant in the fruit rind or exocarp^18,19^. Recently, the enhancement of the antimicrobial activity of PRE has been explored by the co-application of Zn (II), with significant synergistic (potentiated) virucidal activity having been found against *Herpes simplex virus* (HSV)^20^ and bactericidal activity against *Micrococcus luteus*^21^, methicillin-resistant *Staphylococcus aureus* (MRSA), *Escherichia coli, Pseudomonas aeruginosa* and *S. epidermidis*^22^. The significance of combination therapy in combating resistance is known, including that for *C. albicans*^23^. As pomegranate extract was previously shown to inhibit *C. albicans* biofilm formation^14^, the aim of this study was to determine if activity against *C. albicans* could be similarly enhanced using PRE and Zn (II) in combination against planktonic and pre-formed biofilms of *C. albicans*. In addition, PRE and PRE and Zn (II) in combination were investigated for their abilities to promote reactive oxygen species (ROS) production and oxidative stress in *C. albicans.*

## Results

### Characterisation of PRE

**Figure 1:**
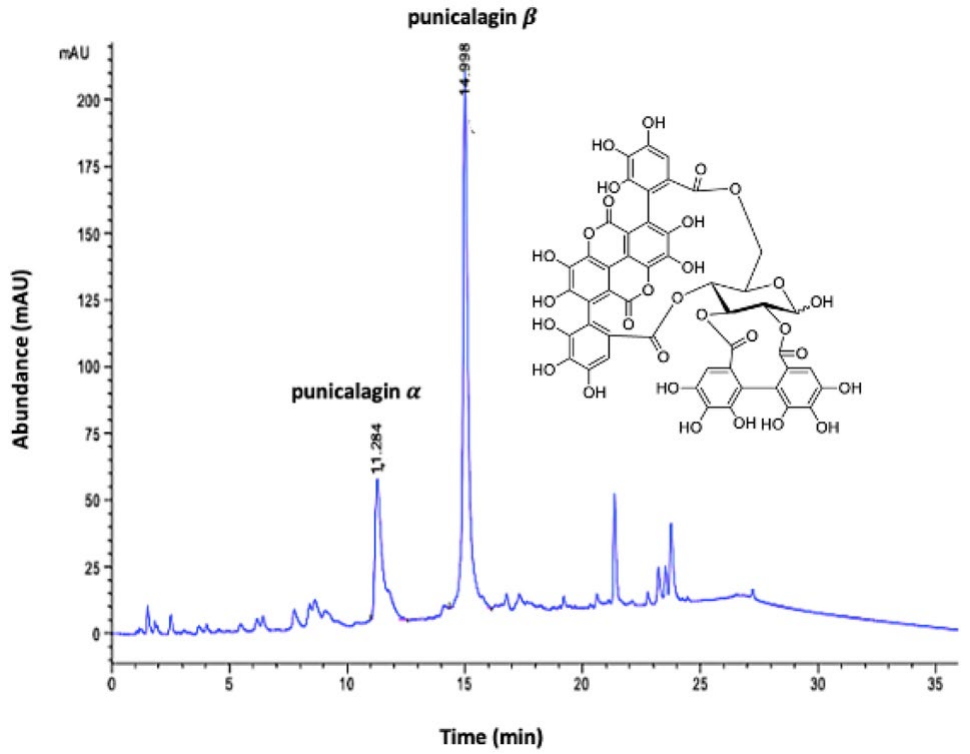
HPLC chromatogram of pomegranate rind extract (PRE) highlighting the major ellagitannin anomers α and β punicalagin; inset: the chemical structure of punicalagin.

Total phenolic content was found as 496 mg of TAE/g of freeze-dried PRE. HPLC chromatogram of PRE showed two major peaks of punicalagin with the equilibrium constant 1.76:1 (K = [B]/[a]), and the amount of punicalagin was determined as 170 mg/g of freeze-dried PRE, using the standard curve obtained with standard punicalagin (Fig. 1).

### Determination of *C. albicans* susceptibility and antifungal interaction of PRE and Zn (II)

**Figure 2:**
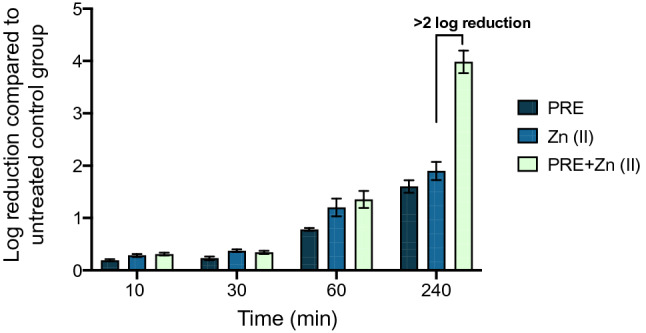
Log reduction of *C. albicans* (ATCC 90028) after incubation of PRE (4 mg/mL), Zn (II) (1.8 mg/mL), and PRE and Zn (II) (4 mg/mL + 1.8 mg/mL) in combination, over a range of contact times (10 min, 30 min, 60 min and 240 min) at 37 °C.

Both PRE and Zn (II) inhibited *C. albicans* growth using the broth dilution assay, with concentrations of 4 mg/mL and 1.8 mg/mL, respectively (Table 1). The antifungal interaction between PRE and Zn (II) was determined as synergistic using the checkerboard assay (FICI = 0.125) (Table 1). This combination also exerted synergistic antifungal activity at 240 min using the time kill assay, which showed more than 2 log reduction compared to PRE or Zn (II) alone (Fig. 2).

**Table 1:** MIC and FICI values for PRE and Zn (II) alone and in combination against *C. albicans* (ATCC 90028), determined by broth dilution and checkerboard method.

	MIC	MIC in combination	FIC	FICI
PRE (mg/mL)	4 ± 1.8	0.25 ± 0.115	0.0625 ± 0.03	–
Zn (II) (mg/mL)	1.8 ± 0.75	0.112 ± 0.05	0.0625 ± 0.03	–
PRE + Zn (II)	–		–	0.125 ± 0.05 (synergism)

### Confocal microscopy and COMSTAT2 analysis of *C. albicans* biofilms in the presence of PRE, Zn (II) and PRE/Zn (II) combined

**Figure 3:**
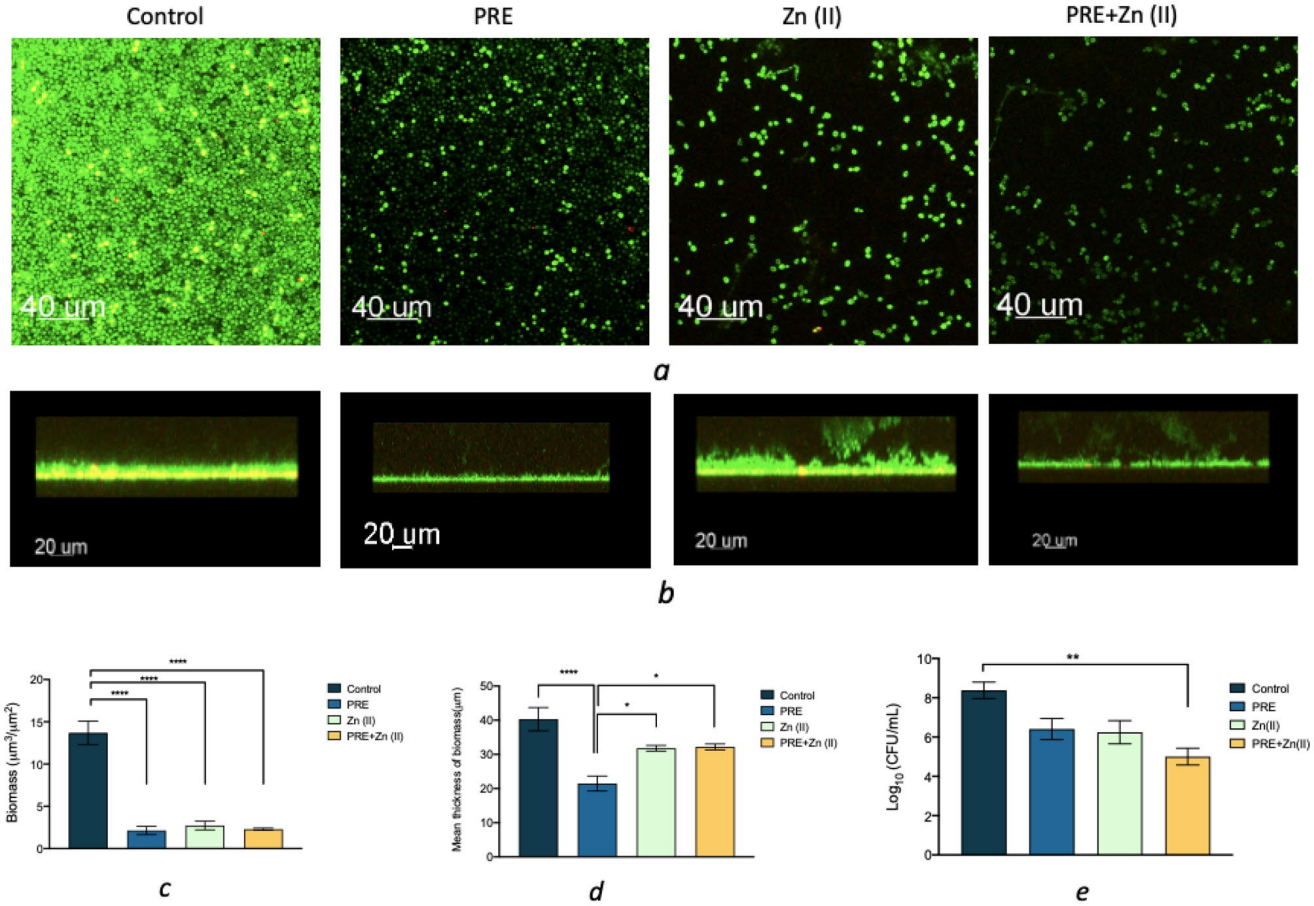
(**a** and **b**) CLSM images of *C. albicans* biofilm after incubation with PRE (4 mg/mL), Zn (II) (1.8 mg/mL) and PRE and Zn (II) combined (4 mg/mL + 1.8 mg/mL), at 37 °C for 24 h. (**c** and **d**) Comparison of different variables of *C. albicans* biofilm using CLSM/COMSTAT2 analysis. (**e**) Log_10_ values of *C. albicans* biofilm a after incubation with PRE, Zn (II) and PRE and Zn (II) combined, at 37 °C for 24 h. Significance indicated by *, where *p < 0.05, **p < 0.01, ***p < 0.001, and ****p < 0.0001*.* Data represented as mean ± SEM (n = 3).

The effects of PRE, Zn (II) and PRE and Zn (II) combination on pre-formed *C. albicans* biofilms were evaluated by confocal microscopy, with quantitative analysis performed using COMSTAT2 analysis. Control cultures (without treatment) formed a dense biofilm (biomass ≃ 15 µm^3^/µm^2^ and mean thickness ≃ 40 µm). A substantial decrease in biomass was observed in the presence of PRE, Zn (II) and PRE and Zn (II) combined (all *p* < *0.0001*). However, a significant reduction in biofilm thickness was only observed in the presence of PRE, compared to both the untreated controls (p < 0.0001) and other treatment groups, Zn (II) and PRE/Zn (II) (both *p* < *0.05*) (Fig. 3).

### ROS production by *C. albicans* in the presence of PRE, Zn (II) and PRE and Zn (II) combined

**Figure 4:**
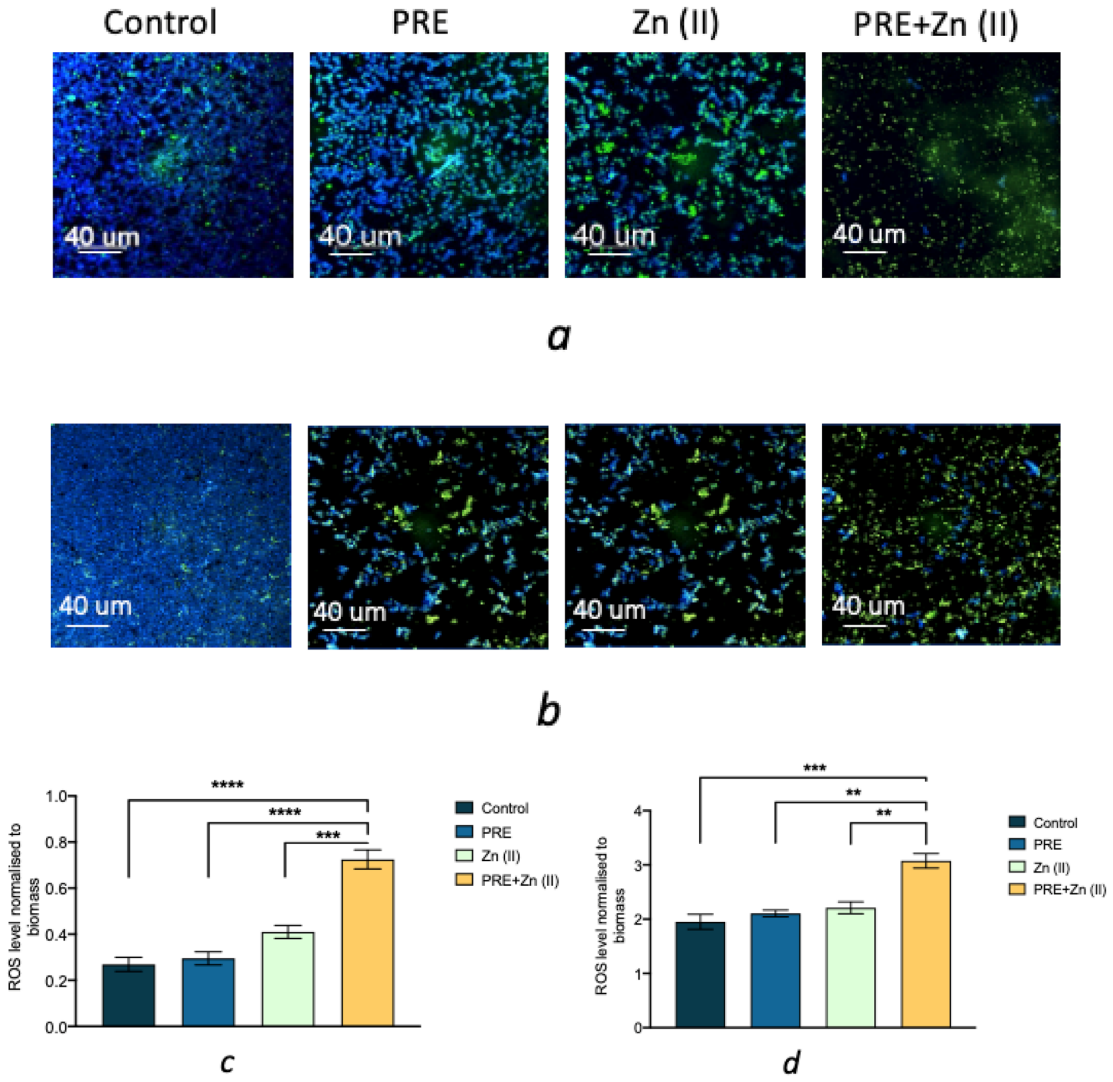
CLSM images of *C. albicans* in planktonic (**a**) and biofilm form (**b**) incubated with PRE (4 mg/mL), Zn (II) (1.8 mg/mL) and PRE and Zn (II) combined (4 mg/mL + 1.8 mg/mL) for 24 h. Blue channel shows calcofluor in *C. albicans* cells walls and green channel shows ROS production in *C. albicans* cells. Graphs shows ROS levels for *C. albicans* in planktonic form (**c**) and biofilm form (**d**) normalised to total biomass. Significance indicated by *, where **p < 0.01, ***p < 0.001, and ****p < 0.0001. Data represented as mean ± SEM (n = 3).

ROS production was measured in planktonic and biofilm forms of *C. albicans* using DCFH-DA, which is hydrolyzed to DCF by intracellular esterases producing a high intensity green fluorescence. Fluorescence intensity was quantified via COMSTAT2 and normalized to total fungal biomass. ROS production was observed in all experimental groups, under both planktonic and biofilm conditions, although *C. albicans* biofilms resulted in higher ROS production than its planktonic equivalents (Fig. 4a,b). PRE and Zn (II) alone applications caused higher ROS generation, compared to untreated controls. However, PRE and Zn (II) in combination caused a substantial increase in ROS production by *C. albicans*, compared to untreated controls and the PRE and Zn (II) alone treatments, under both planktonic (Fig. 4c) and biofilm conditions (Fig. 4d) normalised to total biomass.

### Hyphal growth of *C. albicans* biofilms in the presence of PRE, Zn (II) and PRE and Zn (II) combined

**Figure 5:**
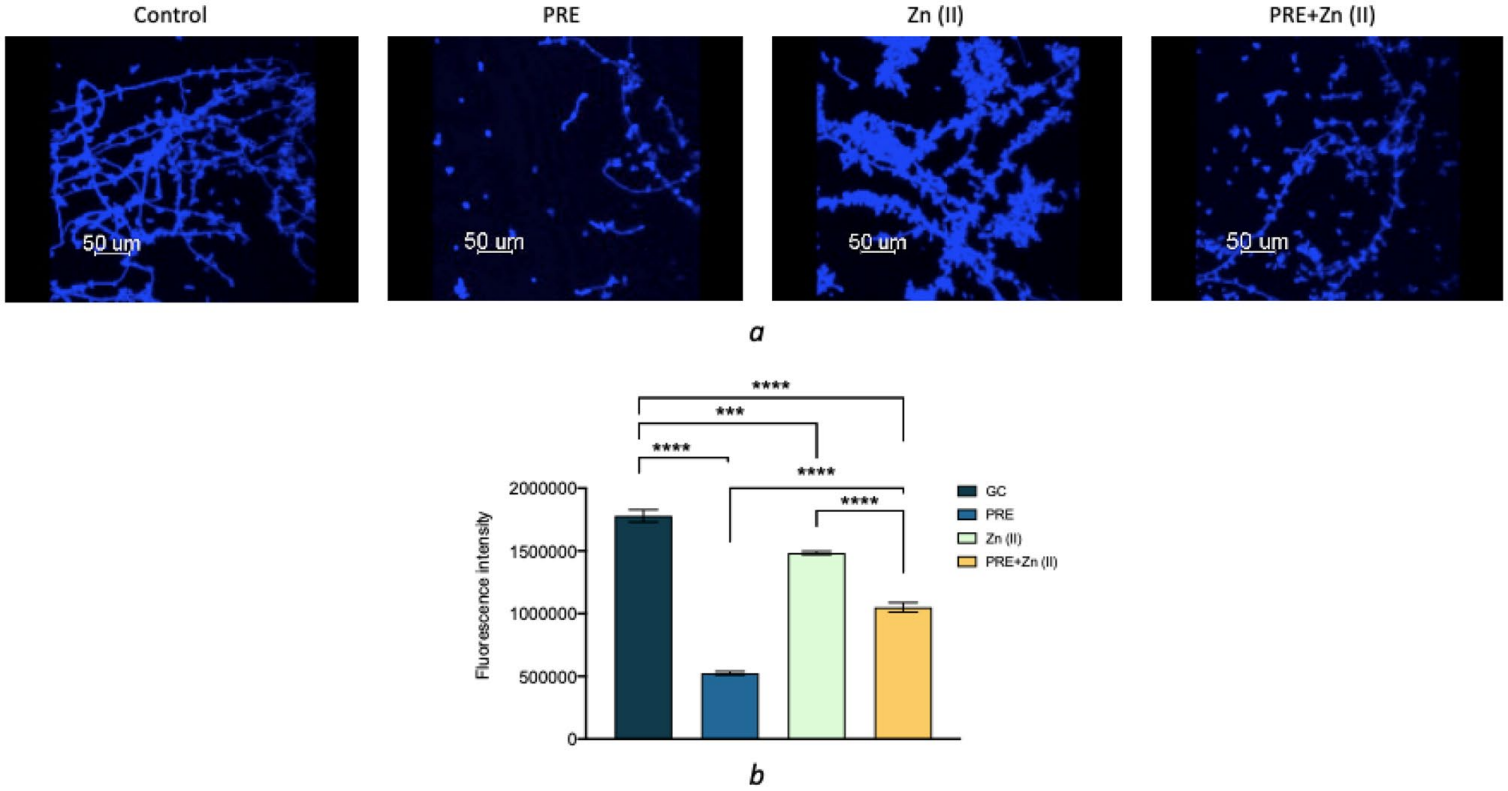
(**a**) CLSM imaging of PRE (4 mg/mL), Zn (II) (1.8 mg/mL) and PRE and Zn (II) combined (4 mg/mL + 1.8 mg/mL) treated and untreated *C. albicans* biofilms, stained with Calcofluor-White to identify hyphal formation (for *C. albicans* cell walls in blue, scale bar = 50 µm). (**b**) The fluorescence intensities of Confocal Microscopy images. Significance indicated by *, where ***p < 0.001, and ****p < 0.0001. Data represented as mean ± SEM (n = 3).

PRE showed the highest inhibitory activity against *C. albicans* hyphal growth (Fig. 5a). While PRE and Zn (II) in combination caused an inhibition in hyphal growth, this inhibitory effect was substantially lower than PRE alone. The fluorescence intensities were significantly reduced in the presence of PRE, Zn (II) and PRE and Zn (II) combined, compared to untreated controls (p < 0.001 for Zn (II) vs untreated controls and p < 0.0001 for PRE and PRE + Zn (II) vs untreated controls). However, PRE exhibited the lowest fluorescence intensities, with significant differences between biofilms treated with PRE, versus the untreated control and other treatments (p < 0.0001) (Fig. 5b).

## Discussion

The frequency of mucosal and systemic *Candida* species infections has increased in recent years, due to various factors including the increasing number of immunocompromised patients, illnesses, and widespread use of broad-spectrum antibiotics^24^. The increasing rate of drug resistance and the low number of antifungal drugs in clinical development highlights the difficulty in treating *C. albicans* infections^25^. As a result, the need for new antifungal compounds has become a major priority worldwide^26^.

Pomegranate has a long history in the treatment of microbial infections and recently, pomegranate extracts have been reported to possess antimicrobial, anti-inflammatory and wound healing activities^27–31^. Pomegranate extract exerted a broad-spectrum antimicrobial activity against both Gram-positive and Gram-negative bacteria, viruses, protozoa, in addition to fungal strains, including *C. albicans*^20,31,32^. Beneficial health activities of pomegranate extracts have been largely attributed to high amounts of hydrolysable tannins, especially punicalagins, punicalin, and ellagic acid^33–36^. These hydrolysable tannins have been shown to precipitate proteins present in the cell membrane which causes disruption of the cytoplasmic membrane, inhibition of nucleic acids, limitation of energy metabolism, cell lysis and death^33,37^. In this study, *C. albicans* was found to be susceptible to PRE and Zn (II) with MIC values of 4 mg/mL and 1.8 mg/mL, respectively. *C. albicans* can grow in three different morphological forms: budding yeast, pseudohyphal and hyphal. This morphological adaptivity gives *C. albicans* an edge in the formation of biofilms on medical device surfaces, resulting in biofilm-associated illnesses^38^. We also evaluated the antibiofilm activity of PRE, Zn (II) and PRE and Zn (II) in combination, via Live/Dead staining analysis by Confocal Microscopy. Microscopy images and quantitative COMSTAT2 analysis demonstrated the disruptive effects of PRE, Zn (II), and PRE and Zn (II) in combination on *C. albicans* biofilms. *C. albicans* generated high-density biofilms in the absence of test compounds and their combination. Only PRE induced significant decreases in the mean thickness of *C. albicans* biofilms, despite all treatments resulting in large reductions in biofilm biomass. In this study, we further investigated the influence of PRE, Zn (II) and PRE and Zn (II) in combination on the morphological forms of the *C. albicans* biofilm using calcofluor white staining. PRE was the most efficient compound at inhibiting hyphae formation, demonstrating similar responses to its effect on *C. albicans* biofilm mean thickness, as *C. albicans* hyphal formation would contribute to biofilm thickness overall. However, only PRE and Zn (II) in combination caused a substantial reduction in the CFU of *C. albicans* cells in biofilm form. This could be result of synergistic fungicidal action of PRE and Zn (II) on *C. albicans* cells, as this combination showed a synergistic antifungal activity in both checkerboard and time-kill assays. Previously, PRE and Zn (II) in combination showed a synergistic antimicrobial activity in vitro against a range of bacteria and HSV^20–22^. PRE and copper combination exhibited an enhanced antimicrobial activity against *E. coli, P. aeruginosa* and* P. mirabilis*^39^. The synergistic antifungal activity found in the current study has great importance as combinational antimicrobial therapy of *C. albicans* infections results in lower toxicity and decreased rate of drug resistance^40^. Similarly, vanillin complex (a phenolic molecule) coupled with various metal ions has previously potentiated antibacterial efficacy against *S. aureus, E. coli, K. pneumoniae, P. aeruginosa*, and *C. albicans*^41^.

The mechanism for the synergistic antimicrobial activity of pomegranate extracts and metal salts is not fully understood, but suggested mechanisms involve the phenolic chemicals forming a complex with metal ions, which has increased antimicrobial action^42^. Also, interaction on the microbe surface could disrupt efflux pump activity, or tannin/Zn endocytosis facilitated where a low pH is employed such that surface charge and electrostatic repulsion is reduced^21^. However, the time kill data alludes to a diffusional aspect as the synergy is not observed in this work until 240 min contact time.

Phenolic compounds, such as those found in pomegranate, are generally known for antioxidative activities^43–45^. However, phenolic compounds can also exhibit pro-oxidative behaviour, depending on such factors as concentration and the intra-cellular environment, including the presence of metal ions at the site of free radical generation. It is claimed that whether phenolic compounds act as antioxidants or pro-oxidants is influenced by the presence of transition metal ions, e.g. iron, copper and zinc^46^. For instance, pro-oxidative activity has been reported for curcumin with copper. In the presence of copper, curcumin showed an apoptosis on cancer cells by increasing secretion of ROS molecules^47–49^. Thus, a possible mechanism behind the synergistic antifungal activity of PRE and Zn (II) combined was evaluated, in terms of the oxidative stress responsiveness of *C. albicans*. In the current study, it was shown that both PRE and Zn (II) did not induce the ROS production of *C. albicans* cells, when they were applied alone and they did produce a similar level of ROS in both planktonic and biofilm level, compared to untreated controls. However, the combination of PRE and Zn (II) was shown to substantially elevate ROS production, compared to untreated controls and when PRE and Zn (II) were applied alone—these high levels would be expected to be harmful to the cells, causing death.

It has been reported that compounds that induce ROS production could act as promising antifungal agents^50^. ROS have apoptotic effects on different cell types, including *C. albicans*, and studies have reported ROS-induced *C. albicans* apoptosis in the presence of acetic acid, resveratrol, farnesol, and antimicrobial peptides^51–54^. In addition to their target specific actions, the fungicidal activity of routinely used antifungal drugs, such as azoles, has been linked to their increased ROS effects^50^. Furthermore miconazole-tolerant *Candida* cells have a high amount of ROS inactivating activity^55^. *C. albicans* have enzymatic and non-enzymatic antioxidant defence mechanisms, such as superoxide dismutase (SOD), that plays a key role in *C. albicans* virulence^56,57^. This elevated ROS level could be a result of the inhibition of *C. albicans* endogenous antioxidant system or the pro-oxidant activity of PRE and Zn (II) in combination. ROS can affect a wide range of biological molecules, including nucleic acids, proteins, and lipids^58^. The pro-oxidative characteristics of PRE and Zn (II) in combination may inhibit mitochondrial respiration enzymes, including NADH oxidase and succino-oxidase^59^. However, pinpointing the particular processes responsible for the antifungal efficacy of this combination against *C. albicans* is difficult, and more research is needed to have a better understanding of the role of ROS in the synergistic antifungal activity of PRE and Zn (II).

There are a limited number of effective antifungal medications commonly used, with new treatments against fungal infections urgently needed. Natural products, in particular polyphenols, are becoming more appealing as an alternative agent in the treatment of infectious disorders, because of their broad-spectrum antibacterial and antifungal effects, low toxicities and low costs^60,61^. In addition, combination drug therapy is known to inhibit resistance mechanisms.

To conclude, this work successfully demonstrated that the polyphenol-rich composition of PRE and the addition of Zn (II), which is already known for its synergistic antibacterial, antiviral and anti-inflammatory properties, could also make this combination a promising novel treatment for *C. albicans* infections^15,20–22,29^. Taken together with previous results, the results reported herein against *C. albicans* supports the development of a novel broadspectrum anti-infective system.

## Materials and methods

### Materials

Pomegranates (of Spanish origin) were obtained from a local supermarket, therefore collection of plant material, complied with relevant institutional, national, and international guidelines and legislation. Zinc sulphate heptahydrate (ZnSO_4_·7H_2_O) and potassium hydrogen phthalate were obtained from ThermoFisher Scientific (Loughborough, UK). Mueller–Hinton broth (MH broth), Mueller–Hinton agar (MH agar), brain–heart infusion agar (BH agar), brain–heart infusion broth (BH broth) were obtained from Oxoid Ltd. (Basingstoke, UK) and Saboraud dextrose 4% agar (SDA) was obtained from VWR chemicals (Leuven, Belgium). Live/Dead Baclight™ Bacterial Viability Kit was obtained from Invitrogen Molecular Probes (Paisley, UK). Calcifluor white stain and DCFH-DA probe were obtained from Sigma-Aldrich (Poole, UK).

### Preparation of pomegranate rind extract (PRE)

Fresh fruits of pomegranate were washed (*Punica granatum* L., Spanish origin) and pomegranate rind separated from its arils. The rind was cut to approximately 2 cm^2^ pieces with a scalpel, before blending in deionised water (25% w/v). The homogenous rind solution was boiled for 10 min, centrifuged 4 times (5980×*g* at 4 °C for 30 min, Heraeus Multifuge 3S/3S-R centrifuge), and filtered through a Whatman 0.45 µm nylon membrane filter. The supernatant was collected, freeze-dried, and then stored at − 20 °C until stock solutions were prepared in pH 4.5 phthalate buffer^62^. The total phenolic content of PRE was found using Folin-Ciocalteu assay with the method described before and the result was shown as tannic acid equivalents (TAE) per gram of freeze-dried PRE^63^. The punicalagin amount in PRE was determined by high pressure liquid chromatography according to the method of Seeram et al.^64^.

### Media used and growth condition of *Candida albicans*

*Candida albicans* was grown on SDA for 24 h at 37 °C. Few colonies from the agar plate were propogated in MH broth and the turbidity of *C. albicans* cells were adjusted with MHB to optical density (600 nm) value of 0.1. Then cell suspension finally diluted with MH broth obtain approximately 1 × 10^6^ cells/mL of *C. albicans* to use in the experiments.

### Anti-*Candida* susceptibility of PRE and Zn (II)

Broth dilution assays were performed to find the minimum inhibitory concentration (MIC) of PRE and Zn (II). *C. albicans* (1 × 10^6^ cells/mL) in MHB were incubated with equal volume of serially two-fold diluted tested agents aerobically at 37 °C for 24 h. The concentrations that caused no turbidity were subsequently determined as MIC values^65,66^.

### Chequerboard assay

Antimicrobial activity interaction between PRE and Zn (II) was determined using the chequerboard assay^67^. Eight doubling dilutions of PRE and Zn (II) were prepared in MH broth and combined (final volume 100 µL) and diluted tested agents were inoculated with *C. albicans* at a density of 1 × 10^6^ cells/mL in each well. Experimental plate was incubated for 24 h at 37 °C in ambient air. The fractional inhibitory concentration index (FICI) for PRE and Zn (II) were calculated using the formula:

$$ {\text{FIC}}_{{{\text{PRE}}}} + {\text{FIC}}_{{{\text{Zn}}({\text{II}})}} = {\text{FICI}} $$

where FIC_PRE_ is the MIC of PRE in combination/MIC of PRE alone, and FIC_Zn(II)_ is the MIC of Zn (II) in combination/MIC of Zn (II) alone. The FICI was interpreted as synergy where FICI ≤ 0.5; no interaction where FICI > 0.5 ≤ 4; or antagonism where FICI > 4^68^.

### Time-kill assay

PRE, Zn (II) and PRE in combination with Zn (II) were investigated for their fungicidal and time-killing activities by measuring viable cell counts. *C. albicans* (1 × 10^6^ cells/mL) was incubated with PRE (2 mg/mL and 4 mg/mL), Zn (II) (0.9 mg/mL and 1.8 mg/mL) and PRE in combination with Zn (II) (2 mg/mL PRE with 0.9 mg/mL Zn (II) and 4 mg/mL PRE with 1.8 mg/mL Zn (II)) at 37 °C. The number of viable cells were obtained by colony counting at specified timepoints (10, 30, 60 and 240 min)^69^. The results were presented as the mean values number of colony forming units (CFU) of quadruplicate measurements from three independent experiments.

### Effects on *C. albicans* biofilms via confocal microscopy

The effects of PRE, Zn (II), and PRE and Zn (II) combined on pre-existing 24 h *C. albicans* biofilms was investigated using a Live/Dead BacLight™ Bacterial Viability test (Greiner Bio One Ltd., Stonehouse, UK). This assay was performed based on the method previously described by Powell et al.^70^. Briefly, *C. albicans* biofilms were generated in a glass-bottomed, 96-well plates by inoculating 100 µL of *C. albicans* at a density of 1 × 10^8^ cells/mL per well. After aerobic incubation at 37 °C for 24 h, the supernatant was replaced with PRE (MIC), Zn (II) (MIC) and PRE in combination with Zn (II) (MIC + MIC) for another 24 h incubation. According to the manufacturer's instructions, each plate was subsequently stained with the Live/Dead BacLight™ Bacterial Viability Kit and observed using the Leica TCS SP5 Confocal Microscope (Leica Microsystems Ltd., Milton Keynes, UK). Images were captured with an 63× oil objective and a 1 µm z-step size. COMSTAT2 plugin with ImageJ analysis software, Version 2.1.0 (US National Institutes of Health, Bethesda, Maryland, USA)^71^ was used to analyse Z-stack pictures produced using Bitplane’s Imaris Programme (Concord, MA, USA). Results were expressed as mean ± SEM (n = 12).

### Reactive oxygen species (ROS) production by *C. albicans* during and following biofilm formation

The intracellular ROS production of *C. albicans* was determined by CLSM, using dichloro-dihydrofluorescein diacetate (DCFH-DA) in a glass bottomed, 96-well plates during and following biofilm formation^72^. *C. albicans* biofilms were developed as described above and ROS production during biofilm formation was assessed by incubating *C. albicans* cells with PRE, Zn (II) and PRE in combination with Zn (II) aerobically at 37 °C for 24 h. Then, each supernatant was discarded and the plates were first stained with 5 µL of Calcofluor-White (0.05% v/v, Sigma-Aldrich) for 1 min, and excited at 355 nm. Then, 15 µL of 10 µM of DCFH-DA was added and incubated for 15 min in the dark at room temperature, excited at 488 nm, and stained plates were visualised with a Leica TCS SP5 Confocal Microscope (Leica Microsystems Ltd, Milton Keynes, UK). Images were obtained with 60 × 1.8 oil objective with a z-step size of 1 μm. The randomly selected fields of view were analysed using COMSTAT2 software with the NIH-ImageJ analysis software for biomass (μm^3^/μm^2^) of two channels, blue for *C. albicans* cell wall, and green for ROS. The ROS level was normalised to total biomass of Calcofluor-White *C. albicans* and presented in a graph as mean ± SEM (n = 12).

### Effects on *C. albicans* hyphal growth

*Candida albicans* biofilms (500 µL of *C. albicans* cells incubated aerobically at 37 °C for 24 h) were formed in 24-well glass bottomed plates. After incubation, biofilms were treated with 0.5 mL of PRE, Zn (II) and PRE in combination with Zn (II) or untreated for growth control for 24 h. Biofilms were washed with phosphate buffered saline (pH 7.2) and stained with 0.05% (v/v) Calcofluor White for 1 min in the dark and visualised with CLSM. Images were analysed using ImageJ analysis software and the results presented as mean ± SEM of fluorescence intensity^73^.

### Statistical analysis

All experiments were performed in triplicate, using independent microbial cultures for all antimicrobial assays. The results were analysed and graphically presented using GraphPad Prism 8.0 software. The one-way ANOVA test with post-test Tukey correction was used and *p* < 0.05 was considered statistically significant.

## Data Availability

The datasets generated during and/or analysed during the current study are available from the corresponding author on reasonable request.
